# Sleep Problems in Parkinson's Disease

**DOI:** 10.1155/2015/507948

**Published:** 2015-12-14

**Authors:** Koichi Hirata, Birgit Högl, Eng King Tan, Aleksandar Videnovic

**Affiliations:** ^1^Department of Neurology, Dokkyo Medical University, Tochigi 321-0293, Japan; ^2^Department of Neurology, Innsbruck Medical University, Innsbruck, Austria; ^3^Department of Neurology, Singapore General Hospital, Singapore; ^4^Department of Neurology, Massachusetts General Hospital, Division of Sleep Medicine, Harvard Medical School, Boston, MA, USA

In his famous monograph “An Essay in Shaking Palsy,” James Parkinson provided astute descriptions of impaired sleep in his case series of patients with Parkinson's disease (PD) two centuries ago. It is only three decades ago that sleep dysfunction started to attract attention of medical and scientific communities involved in the clinical care and research of PD. Tremendous advancements in our understanding of impaired sleep and alertness associated with PD have developed since then.

Sleep problems are one of the major, challenging issues in patients with PD, affecting a significant number of patients. The etiology of sleep problems is multifactorial, including disease-related nocturnal symptoms, medication adverse effects, and primary sleep disorders including restless legs syndrome, rapid eye movement sleep behavior disorder, and sleep apnea syndrome. These causes of impaired sleep-wake cycles in the PD population often coexist or even overlap, rendering the management of sleep problems in PD patients difficult. Also, excessive daytime sleepiness is observed in a significant number of patients. In PD patients, therefore, appropriate assessment of disease-related nocturnal disturbances and primary sleep disorders is imperative. This special issue addresses unmet need for understanding PD-related sleep problems.

PD sleep scale 2 (PDSS-2) is a recently developed tool for screening and managing sleep disturbance in PD patients, consisting of 15 items which are clinically relevant to nocturnal problems including nonmotor and motor problems in PD [1]. The original PDSS-2 (German and English) has been translated into several languages, including Japanese [2] and Italian [3]. The PDSS-2 has been used to observe treatment response. In a double-blind, placebo-controlled trial, including 287 PD patients, mean PDSS-2 total score decreased by −5.9 points with rotigotine and by −1.9 points with placebo [4]. In this special issue, K. Horváth et al. estimated the threshold representing minimal clinically important difference of the PDSS-2 total score: the study results showed −3.44 points for detecting improvement or the threshold of 2.07 points for observing worsening. This finding is important when planning studies using the PDSS-2 as outcome measures.

Full-night polysomnography (PSG) is a gold standard for diagnosing sleep apnea syndrome; however, applying PSG is often difficult in patients with PD, who have severe parkinsonism or psychiatric comorbidity. P. Gros et al. evaluated the usefulness of unattended portable monitoring (PM) for diagnosis of obstructive sleep apnea (OSA) in patients with PD. Although discrepancy between portable monitoring and PSG was greater in PD patients with more motor dysfunction, the authors confirmed the usefulness of portable monitoring in diagnosing moderate to severe OSA in PD patients. These results provide the rationale for the use of portable sleep monitoring in PD and are very relevant for circumstances where a complete PSG in a sleep laboratory is not available and/or feasible.

M. Kaminska et al. performed a review on the relationship between OSA and PD. Although the clinical significance of OSA in PD has been controversial [5, 6], the authors suggest the possibility that treatment of OSA could delay cognitive decline or motor dysfunction in patients with PD. This area of research is of high significance as it is important to assess the prevalence of OSA and the impact of its treatment in the PD population.

D. Martinez-Ramirez et al. correlated PSG findings and sleep disorders with clinical characteristics in PD patients and found that sleep disorders and sleep architecture were poorly predictable by clinical characteristic of PD patients. This comprehensive study demonstrates the complexity of sleep dysfunction associated with PD and its complex associations with metrics of PD.

In a fine and thoughtful study K. Suzuki et al. provided an interesting insight into the complex relationship between PD and restless legs syndrome (RLS) and leg motor restlessness (LMR). A significant relationship between RLS and PD is suggested by observing a favorable response to dopaminergic treatment in both disorders. However, RLS prevalence in PD patients varies according to different studies. Recently, LMR, characterized by an urge to move the legs that does not fulfill the diagnostic criteria for RLS, has been reported more frequently in patients untreated with PD than healthy controls. This review article may provide a new insight into the relationship between RLS, LMR, and PD.

Finally, the complex and still only partially understood interaction of impulse control disorders in PD and sleep is discussed in a review from the clinic of one of the guest editors.

In summary, sleep problems are common but underreported by PD patients and underdiagnosed by health professionals. Further understanding of mechanisms that underlie impaired sleep and alertness in PD will allow for development of much needed treatment approaches for this nonmotor manifestation of PD.

*Koichi Hirata* *Koichi Hirata* *Birgit Högl* *Birgit Högl* *Eng King Tan* *Eng King Tan* *Aleksandar Videnovic* *Aleksandar Videnovic*
